# A Case Report of Overcoming an Obstructive, Pedunculated Cervical Fibroid at the Time of Uterine Evacuation

**DOI:** 10.1155/2019/2651680

**Published:** 2019-06-09

**Authors:** Jayesh Tigdi, Cynthia Chan

**Affiliations:** ^1^Department of Obstetrics & Gynecology, McMaster University, Hamilton, Ontario, Canada; ^2^Department of Obstetrics & Gynecology, Western University, London, Ontario, Canada

## Abstract

**Background:**

Fibroids, which can impact pregnancies at later gestations, such as obstructing delivery, may also affect the pregnancy termination process.

**Case:**

We present the case of a 28-year-old G1 at 18 weeks who consented for a genetic pregnancy termination via dilation and evacuation. During the typical preparatory procedure with laminaria, it was noted that a 5-6cm cervical fibroid prolapsed into the vagina obstructing access to the uterine cavity. Through osmotic dilation followed by cervical Foley catheter ripening, a planned myomectomy was possible with minimal blood loss prior to uterine evacuation.

**Conclusion:**

Through appropriate counselling, planning, and effective cervical dilatation, a planned myomectomy for prolapsing fibroids at the time of termination of pregnancy is possible. This can prevent unnecessary hysterotomy and avoid need for subsequent cesarean section.

## 1. Introduction

A number of terminations of pregnancy are for prenatally confirmed fetal genetic diagnoses [1]. Due to the timeline of the routine prenatal genetic screening process, the decision to end these pregnancies tends to be taken further along in gestational age. Therefore, complications of later pregnancy can have an impact on the delivery of care in the process of pregnancy termination.

During evacuation of the uterus, cervical dilatation is important as instrumentation is required to allow passage of contents with reduced cervical trauma and to minimize risk of retained tissue [2]. Indeed, fibroids can impede this passage and, at term, delivery sometimes necessitates hysterotomy. An obstructive cervical fibroid is a known indication for operative term delivery via cesarean section [3]. There are case reports of cervical fibroid posing issues in peripartum care at or near term [4, 5]. However, there is little literature on the management of elective termination for pregnancies complicated by cervical fibroid. In this case report, we describe, in accordance with SCARE criteria [6], an unusual technique of dilatation and evacuation through an obstructive fibroid at an academic centre.

## 2. Case Report

The patient involved was a healthy 28-year-old G1 at 18 weeks of gestation who consented for pregnancy termination via preparatory laminaria dilators and subsequent operative dilatation and evacuation for a confirmed, lethal diagnosis of Trisomy 18. Aside from an ultrasound-diagnosed lower uterine fibroid, her antenatal course was unremarkable and she had had a normal pelvic examination just prior to conceiving.

On preparation of the cervix for dilatation, a speculum exam revealed an obstructing 5-6cm fibroid protruding through the cervix into the vagina. The cervix itself was not visualized due to the size of the fibroid and its protrusion into the vagina. On bimanual exam, the internal os was closed around the fibroid, which appeared to arise from the level of the internal os of the cervix. In order to prepare the cervix for dilation, osmotic dilators were tucked around the fibroid within the cervix. After three hours, the internal os was a finger tip dilated with further ripening required. Rather than a sequential set of dilators which would be challenging to place and maintain around the fibroid due to angulation, a cervical Foley catheter was employed to ripen the cervix further. The intent was to allow proper placement of a dilator without increasing the risk of false passage creation.

Given the obstructive fibroid, the patient consented for a myomectomy prior to the evacuation, with the added, increased risk of hemorrhage. The possibility of an operative hysteroscopy was explained, as it would allow for removal of the stalk of the fibroid in its entirety, as well as cauterization of the base itself should it be required. Prior ultrasound had indicated that the fibroid was within the lower uterine segment, and the location of the base could not yet be identified.

**Figure 1:**
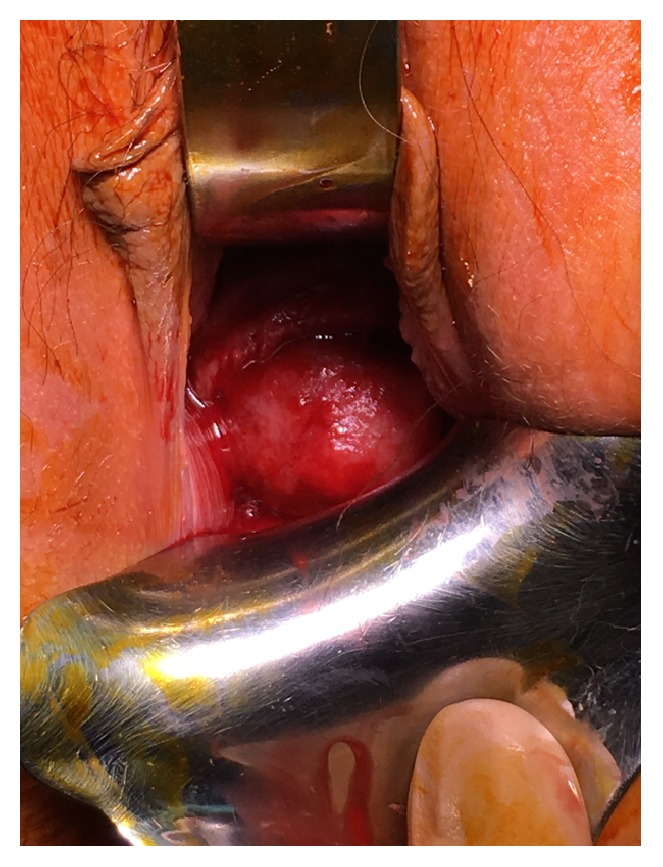
Obstructive cervical fibroid prolapsing through into vagina.

**Figure 2:**
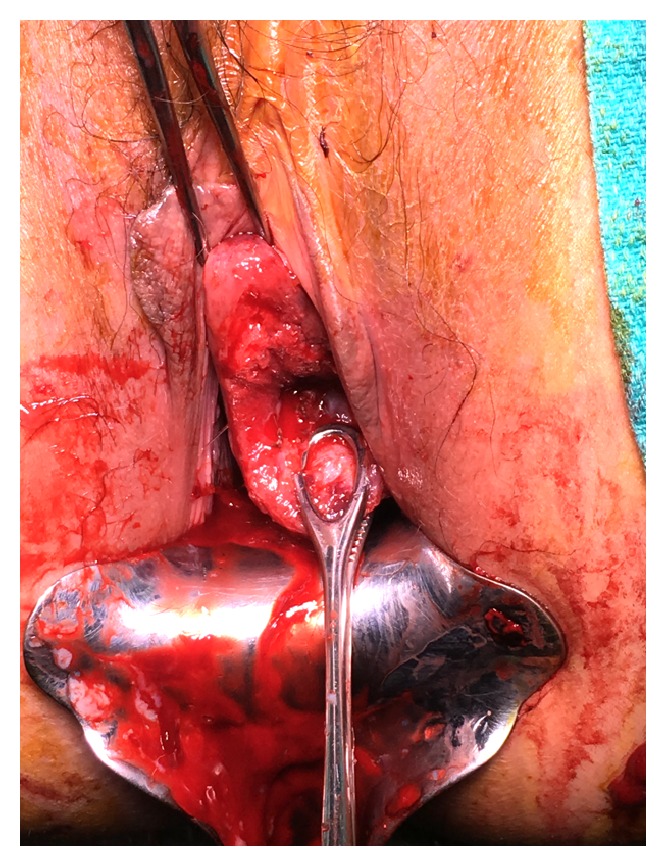
Cervix postmyomectomy with cauterized fibroid stalk base. Ring forceps on posterior lip of cervix and single-toothed tenaculum on anterior surface of cervix.

The following day with the Foley having fallen out and under general anesthetic, examination revealed a sufficiently 3-4cm dilated cervix with the prolapsed fibroid now slightly recessed into the cervix due to the dilatation of the internal os. There was clear visualization of the fibroid stalk originating from within the endocervix (Figure 1). To minimize blood loss intraoperatively, dilute vasopressin (8 units) with 1% lidocaine (20ml) was infiltrated as a paracervical block and at the base of the fibroid. The fibroid was grasped and transected at its base with cautery used to maintain hemostasis. With the obstruction cleared (Figure 2), the remainder of the uterine evacuation was carried forth in the usual manner utilizing a 12mm suction curette. Sharp curettage confirmed that there was no fibroid base remaining. At the conclusion of the case, hemostasis was noted. The patient's postoperative course was uncomplicated.

## 3. Discussion

To our knowledge, this is the first case report of an obstructive fibroid at the time of performing a uterine evacuation for products of conception. Before pregnancy, the patient's fibroid likely remained intrauterine as she had had a Pap smear just prior to pregnancy with no note of any visible abnormalities. With the growing uterus, through mass effect, the fibroid likely prolapsed through the cervical canal, creating a challenge in dilating the cervix for the required procedure.

Although a reportedly more painful procedure, the cervical Foley catheter can be used for cervical ripening when cervical dilators may prove ineffectual. However, to reduce the risk of spontaneous abortion/preterm labour outside the hospital, this is not routine practice [7]. This patient came from a distance, and when performing serial cervical dilatation, our centre requires that patients remain in town after placement of dilators due to the small risk of labour [8].

The cervical Foley was successful in dilating the cervix, as on returning the next day the cervix was dilated with enough access to the uterine cavity and the base of the fibroid stalk was easily visualized. By securing the vascular supply of the fibroid, we were able to successfully perform the myomectomy; with this removed, we had unobstructed access for evacuation of the uterine contents.

In other cases of obstructing fibroids, often hysterotomy is performed rather than attempting a dilation and evacuation because this would allow definitive removal of tissue, rather than having difficulty accessing the tissue and increasing the risk of retained products. However, a hysterotomy is known to have a higher risk of morbidity than dilation and evacuation, in addition to the risk for later pregnancy. Certainly with term pregnancies, an obstructing fibroid is an indication for a cesarean section as it may prevent descent of the fetus [9].

A controlled clinical setting is required to ensure patient safety when performing a myomectomy in pregnancy, as hemorrhage risk is significant [10]. The combination of appropriate patient counselling, assured cervical dilatation with a cervical Foley catheter, and intraoperative blood loss prophylaxis was key in saving this patient from the morbidity of hysterotomy [11]. Further to the patient's benefit, removal of this fibroid may have prevented a cesarean section and other potential complications in a subsequent pregnancy [12].
